# Identification and classification of human cytomegalovirus capsids in textured electron micrographs using deformed template matching

**DOI:** 10.1186/1743-422X-3-57

**Published:** 2006-08-18

**Authors:** Martin Ryner, Jan-Olov Strömberg, Cecilia Söderberg-Nauclér, Mohammed Homman-Loudiyi

**Affiliations:** 1 Department of Medicine, Centre for Molecular Medicine, Karolinska Institutet, Stockholm, Sweden; 2 Department of Mathematics and NADA, Royal Institute of Technology, Stockholm, Sweden

## Abstract

**Background:**

Characterization of the structural morphology of virus particles in electron micrographs is a complex task, but desirable in connection with investigation of the maturation process and detection of changes in viral particle morphology in response to the effect of a mutation or antiviral drugs being applied. Therefore, we have here developed a procedure for describing and classifying virus particle forms in electron micrographs, based on determination of the invariant characteristics of the projection of a given virus structure. The template for the virus particle is created on the basis of information obtained from a small training set of electron micrographs and is then employed to classify and quantify similar structures of interest in an unlimited number of electron micrographs by a process of correlation.

**Results:**

Practical application of the method is demonstrated by the ability to locate three diverse classes of virus particles in transmission electron micrographs of fibroblasts infected with human cytomegalovirus. These results show that fast screening of the total number of viral structures at different stages of maturation in a large set of electron micrographs, a task that is otherwise both time-consuming and tedious for the expert, can be accomplished rapidly and reliably with our automated procedure. Using linear deformation analysis, this novel algorithm described here can handle capsid variations such as ellipticity and furthermore allows evaluation of properties such as the size and orientation of a virus particle.

**Conclusion:**

Our methodological procedure represents a promising objective tool for comparative studies of the intracellular assembly processes of virus particles using electron microscopy in combination with our digitized image analysis tool. An automated method for sorting and classifying virus particles at different stages of maturation will enable us to quantify virus production in all stages of the virus maturation process, not only count the number of infectious particles released from un infected cell.

## Background

Virus assembly is an intricate process and a subject of intensive research[1]. Viruses utilize a host cell to produce their progeny virus particles by undergoing a complex process of maturation and intracellular transport. This process can be monitored at high magnification and resolution utilizing electron microscopy, which allows visual identification of different types of virus particles in different cellular compartments. Important issues that remain to be resolved include the identity of the viral proteins that are involved in each step of this virus assembly process, as well as the mechanism of the underlying intracellular translocation. Localization of different types of virus particles during virus maturation is currently made by hand. Structural aspects of the virus maturation are generally hard to address although visualisation techniques such as tomography and cryo-Electron Microscopy (cryo-EM) have contributed tremendously to the vast information on virus structures. These techniques provide information on stable, often mature virus particles. Genetic tools are available to produce mutants of key viral protein components, and the structural effects can be visualized by electron microscopy (EM). However there is a lack of proper tools to characterize the structural effects, especially intermediate and obscure particle forms and to quantify virus particles properly in an objective way. Image analysis tools to characterize and quantify virus particle maturation and intracellular transport would facilitate objective studies of different virus assembly states employing electron microscopy. A lot of information is acquired when studying virus production by EM, but the data need to be summarized and statistics produced from it in order to evaluate the structural effects and be able to draw conclusions from the study. Extraction of data from images by image analysis will be a valuable tool in virus assembly studies.

Here we describe development of an automated system to assist in the identification of virus particles in electron micrographs. As a model, we have used fibroblasts infected with human cytomegalovirus (HCMV), a virus of the β-herpes class. During infection with human cytomegalovirus, many different intermediate forms of the virus particle are produced[2]. During assembly of the herpesvirus, the host cell is forced to make copies of the viral genetic material and to produce capsids, a shell of viral proteins, which encase and protect the genetic material. Capsids are spherical structures that can vary with respect to size and symmetry and may, when mature be enveloped by a bilayer membrane. The maturation of virus capsids is an important stage in virus particle production, and one that is frequently studied. However, their appearance in electron micrographs varies considerably; making analysis of the virus assembly a challenge. A unique feature of herpesviruses is the tegument, a layer of viral proteins that surround the capsid prior to final envelopment. The envelope is acquired by budding of tegumented capsids into secretory vesicles in the cytoplasm [3]. Thereafter, infectious virus particles exit the host cell by fusion of these virus containing vesicles with the plasma membrane.

**Figure 1:**
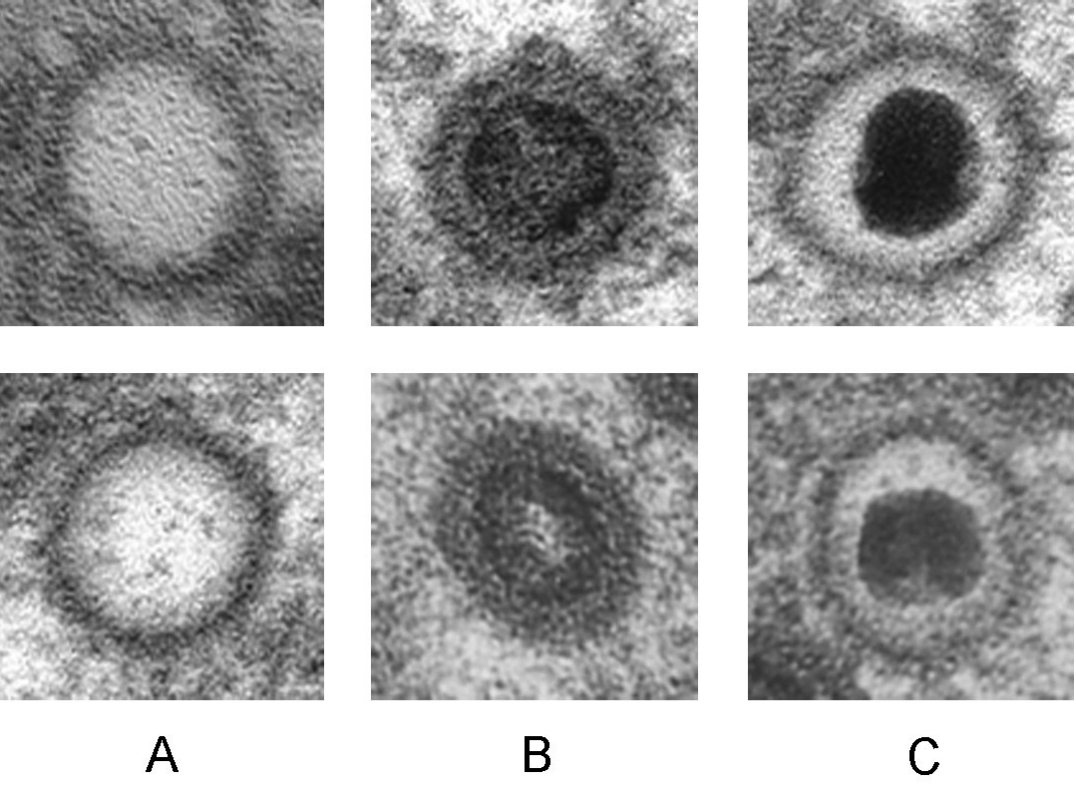
Herpesvirus nucleocapsids at defined stages of maturation. A) Empty nucleocapsids. B) Nucleocapsids with a translucent core. C) Nucleocapsids containing packaged DNA.

**Figure 2:**
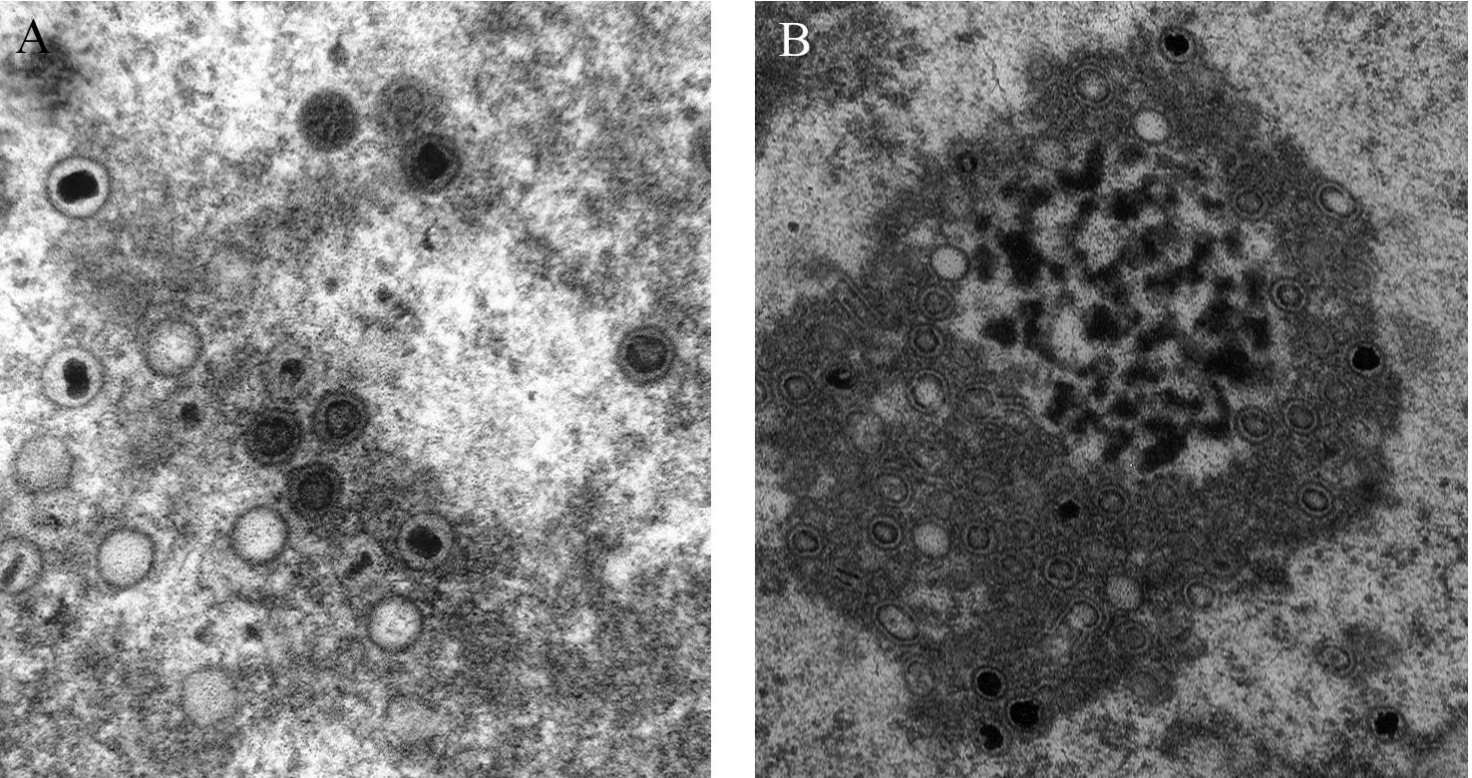
Typical transmission electron micrograph images of developing herpesvirus whose analysis is desirable (A and B). Clearly defined and non-deformed human cytomegalovirus particles (A). Diverse types of background texture and deformed particles in the cell nucleus (B).

Previously we have developed an objective procedure for the classification and quantization of virus particles in such transmission electron micrographs[4]. In the related analysis of cryo-EM images, considerably more effort has been devoted to exploring different methods of identification, as discussed in a recent review[5]. In cryo-micrographs, cross correlation employing multiple templates[6] and methods for edge detection[7] have been applied successfully. Accordingly, in the present investigation, a similar approach has been applied to the analysis of HCMV capsids in the nucleus of infected cells that are at defined states of maturation, i.e., empty capsids (called A), capsids with a translucent core (B) and capsids containing packaged DNA (C), (Figure 1). Suitable approaches allowing characterization and quantification of the maturation of virus particles and their intracellular translocation would facilitate objective studies of these phenomena employing electron microscopy. However, the electron microscope images are difficult to analyze and describe in an objective way because of their heavily textured background. In addition, individual virus particles display a wide variety of shapes, depending on their projection in the electron micrograph, the procedure utilized to prepare samples for electron microscopy and the settings used for photography. Typical electron micrographic images, the analysis of which could provide valuable information are shown in Figure 2.

## Results

### Experimental setup

The standardization and testing were carried out on separate sets of images, two for training and 12 for testing. The number of samples used for standardization was 4, 7 and 10 for the A, B, and C test functions, respectively. The test images contained a total of 53 A capsids (14%), 239 B capsids (64%) and 83 C capsids (22%), and the boundaries of deformation were set at (φ_*R*_, r¯
 MathType@MTEF@5@5@+=feaafiart1ev1aaatCvAUfKttLearuWrP9MDH5MBPbIqV92AaeXatLxBI9gBaebbnrfifHhDYfgasaacH8akY=wiFfYdH8Gipec8Eeeu0xXdbba9frFj0=OqFfea0dXdd9vqai=hGuQ8kuc9pgc9s8qqaq=dirpe0xb9q8qiLsFr0=vr0=vr0dc8meaabaqaciaacaGaaeqabaqabeGadaaakeaacuWGYbGCgaqeaaaa@2E31@, *d*, φ_*D*_) ∈ ([0,2π], [0.83,1.2], [0.83,1.2], [0,2π]).

### The false negative- and false positive ratios

**Figure 3:**
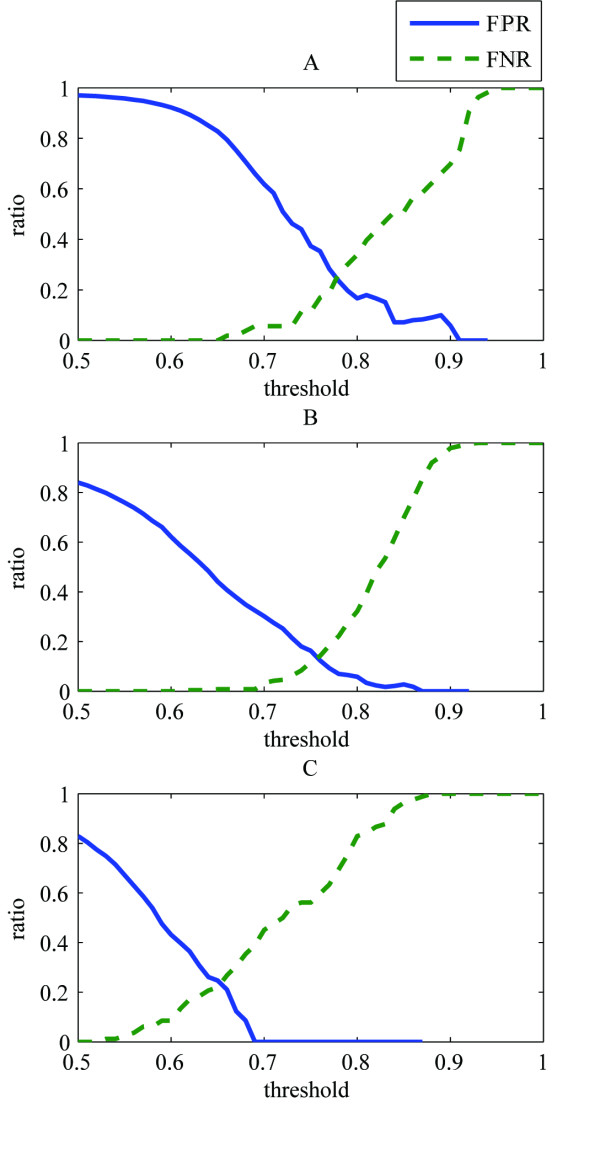
False positive (FPR) and false negative (FNR) ratios for the different test functions A, B and C. The FNR is defined as the ratio between the number of authentic structures rejected incorrectly by the procedure employing a certain threshold value for the matching measure, and the actual number of virus particles present as determined by a virologist. Analogously, the FPR is the ratio between the number of spurious structures identified as being authentic and the total number of structures considered to be authentic by this procedure.

The method was evaluated by comparing our results with those of experienced virologists. The false positive- (FPR) and the false negative ratios (FNR) were calculated as a function of the threshold value for the matching correlation (Figure 3). For comparison with other methods, cross over of the curves occurred at 0.25 for the A test function, 0.13 for the B test function and at 0.23 for the C test function. As described in the introduction, various procedures have been developed to solve the related problem of finding different projections of a particular particle in cryo-EM images for the three dimensional reconstruction of virus particles. Though our method has a different aim, helping in the process of exploring viral maturation instead of finding different projections of a particular particle, our procedure demonstrates similar accuracy with respect to the false negative and false positive ratios.

### Quantification of structures in electron micrographs

**Figure 4:**
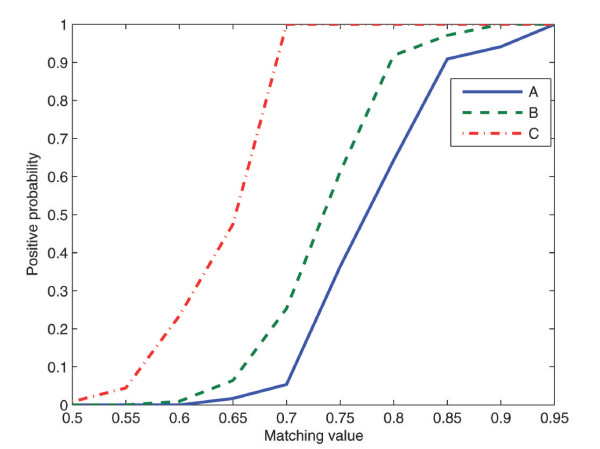
The graph shows the positive probability functions (PPFs) for the test functions A, B and C. The graph depicts the relative frequency of virus particles identified correctly by the procedure at a certain matching value. For comparison an ideal method providing complete separation between true and false structures would result in a Heaviside step function at some threshold value.

**Figure 5:**
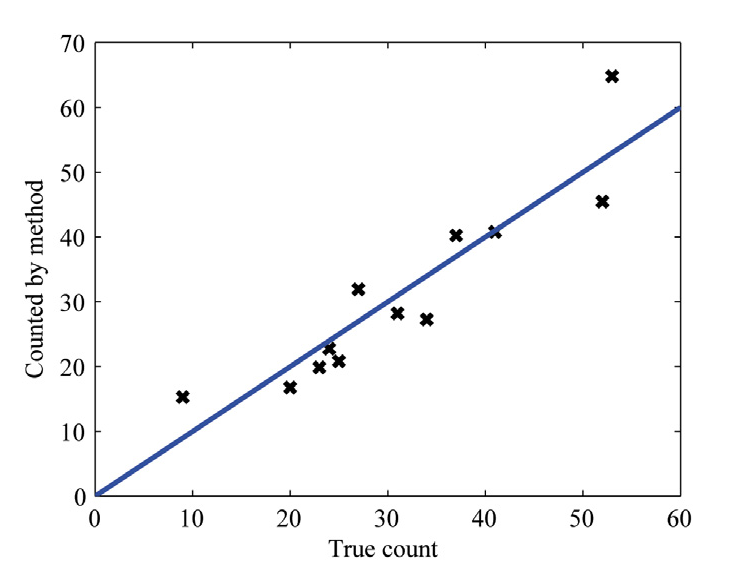
Comparison of the actual total number of viral structures present in a set of test images (X-axis) as determined by a virologist to the number identified by our procedure (Y-axis). The line in this graph depicts the identity function. The mean difference is 0.16 and the standard deviation 5.63. The significance level of the null hypothesis H_0_, i.e., "The mean difference = 0", is 0.92.

The positive probability function (PPF) values calculated from the results presented above are shown in Figure 4. For comparison, an ideal case procedure providing complete separation between true and false structures would result in a Heaviside step function at some threshold value. A scatter plot of the total number of viral particles identified as being present in a set of test images by our procedure in comparison to the correct number as determined by a virologist is shown in Figure 5, together with the identity function. Clearly, there is close similarity between these two values (mean difference = 0.16, standard deviation of 5.63), which in the ideal case would be points on the identity function. The fact that the level of significance of H_0 _was 0.92 according to Student's t-test indicates that there was a fair probability that there was no systematic difference between these two approaches in mean. These results show that fast screening of the total number of viral structures at different stages of maturation in a large set of electron micrographs, a task that is otherwise both time-consuming and tedious for the expert, can be accomplished rapidly and reliably with our automated procedure.

**Figure 6:**
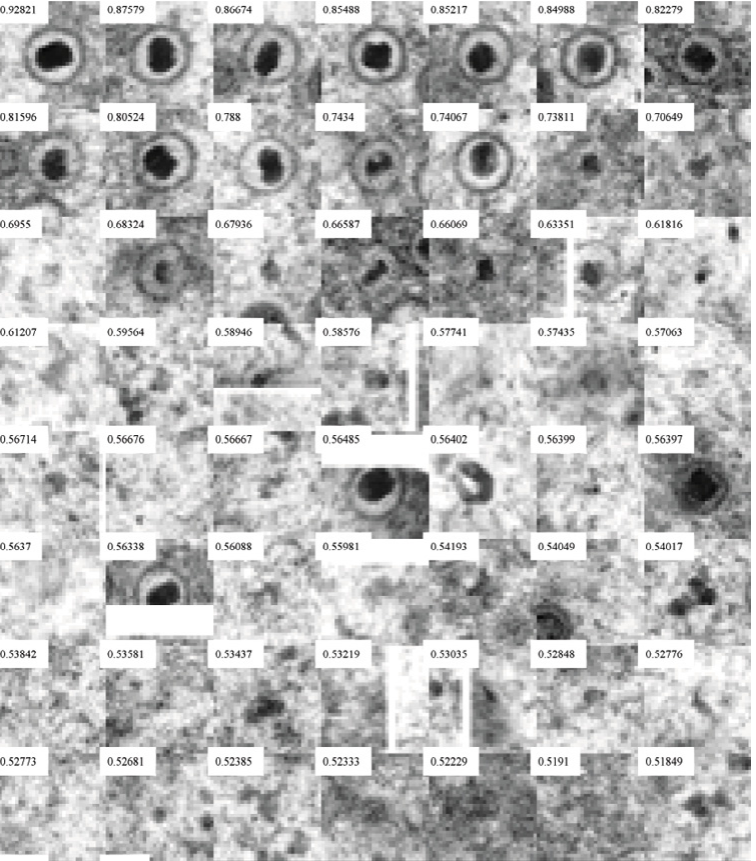
Our procedure allows automated production of a map that identifies locations of interest in an electron micrograph, illustrated here for the C test function. Instead of simply counting and comparing structures in an unprocessed image, the virologist is aided considerably in this task by the availability of such a map. The various structures are sorted left to right in order of descending matching values beginning at the left side of the top row.

On the basis of the set of positions in an image at which structures of interest are located, a map such as that depicted in Figure 6 can be produced, thereby facilitating the manual counting of these structures considerably and also gives a framework for manual analysis.

## Discussion

During the development of this method, several mathematical aspects were examined in more detail. Singular value decomposition (SVD) adds orthogonal dimensions to the test function used here, but resulted in additional information leading to improved segmentation. Use of the actual pixel values at each point of the support can be extended to localized functions, which opens the way for multi-resolution analysis involving wavelets[8] in a sparse and deformable manner. This possibility was explored with the generic Haar wavelet and Daubechies orthogonal wavelets of length 4, 6, and 8. However, since the images employed contain spurious structures at stochastic positions and of various sizes, the use of wavelets did not result in improvement either.

Our procedure described here opens the way for non-uniform deformations, such as independent translation of the points associated with the DNA core inside the capsid. Due to the large computational costs involved, this approach was not tested here, but it could represent an improvement. Methods of deformation analysis that do not employ non-linear programming techniques would be of interest to evaluate in this context. Continuous amplification and suppression of invariant and variant parameters could also be substituted for truncation, thereby allowing weighting of the norms and inner products.

## Conclusion

Monitoring the in-cell structural morphology of virus assembly helps the virologist find novel insights on how to combat the virus infection and develop antiviral strategies. When investigating the process of virus assembly information concerning the structural topology in relationship to the stage of maturation is usually not available or vaguely defined. For this purpose, we have developed a method for the benefit of electron microscopy users, to help gather and quantify structural information on virus assembly from textured electron micrographs. An effective algorithm, as described in this article, has been developed for recognizing profiles of virus particles. Once a few starting points have been obtained by classifying a set of obvious structures, these can be used to expand the set of classified structures by identifying similar structures with the matching function employed. This approach helps make the mapping of virus maturation in electron micrographs rapid, objective, reliable and easy to describe. In this article we describe the method and give an example of how deformable templates can be made and used for matching in micrographs to quantify the existence of intracellular virus particles.

## Methods

### Cell cultures

Human embryonic lung fibroblasts (HF) were maintained in bicarbonate-free minimal essential medium with Hank's salts (GIBCO BRL) supplemented with 25 mM HEPES [4-(2 hydroxyethyl)-1-piperazine ethanesulfonic acid], 10% heat-inactivated fetal calf serum, L-glutamine (2 mM), penicillin (100 U/ml) and streptomycin (100 mg/ml) (GIBCO BRL, Grand Island, NY, USA). The cells were cultured in 175 cm^2 ^tissue culture flasks (Corning, New York, USA) for a maximum of 17 passages.

### Viral infection

The HF cells were infected with HCMV strain AD169 employing a multiplicity of infection (MOI) of 1. The virus containing supernatants were collected 7 or 10 days post-infection (dpi), cleared of cell debris by low-speed centrifugation and frozen at -70°C until used for inoculation.

### Electron microscopy

In order to examine virus-infected cells by electron microscopy, uninfected and HCMV-infected cells were harvested at 1, 3, 5, and 7 dpi and thereafter fixed in 2% glutaraldehyde in 0.1 M sodium cacodylate buffer containing 0.1 M sucrose and 3 mM CaCl_2_, pH 7.4 at room temperature for 30 min. The cells were then scraped off with a wooden stick and transferred to an Eppendorf-tube for continued fixation overnight at 4°C. Following this procedure the cells were rinsed in 0.15 M sodium cacodylate buffer containing 3 mM CaCl_2_, pH 7.4 and pelleted by centrifugation. These pellets were then postfixed in 2% osmium tetroxide dissolved in 0.07 M sodium cacodylate buffer containing 1.5 mM CaCl_2_, pH 7.4, at 4°C for 2 hours; dehydrated sequentially in ethanol and acetone; and embedded in LX-112 (Ladd, Burlington, VT, USA). Contrast on the sections was obtained by uranyl acetate followed by lead citrate and examination performed in a Philips 420 or a Tecnai 10 (FEI Company, Oregon, USA.) transmission electron microscope at 80 kV.

### Image acquisition, discretization and analysis

Electron micrographs of HCMV-infected HF cells were digitalized employing an 8-bit gray scale at a resolution of 5.5 nm/pixel in a HP Scanjet 3970. The implementation was performed with Matlab 7.0.1 (The Mathworks Inc., Natick, MA, USA) and Sun Java 1.4.2 software on a Dell Optiplex GX260 personal computer. This analysis involved an easy-to-use graphical interface and automation of the parameters described below for rapid and convenient use.

### Mathematical outline

Our aim was to develop a user friendly and reliable tool for studies of intracellular virus assembly. Our approach was based on finding a compact set of points in *R*^2^, the field of the micrograph, for each of which a point has a corresponding function value. This set of points and their function values are collectively referred to as a test function or template and can be described by a sequence {(*x*_*k*_, *c*_*k*_)}_*k *_where *x *is the point and *c *is the function value. The test function is produced in such a fashion that the sequence of function value is correlated to the values on the gray scale of the corresponding points. Accordingly, a defined set of virus particles of the same type is required in order to train and design the sequence to provide a template for this specific particle structure. This sparse representation allows facile deformation and adjustments of the template to individual virus particles whose shape in the micrograph is more-or-less elliptical.

### Deformation pre-processing

The positions of the substructures within the same type of viral particles vary in the different images, i.e., the virus particles are sometimes deformed in such manner as to appear in different elliptical forms. In order to create the test functions we utilized linear vector spaces [4], which demands that the vector space positions analyzed are relatively fixed. Uniform linear transformation was chosen to approximate the deformations, since it covers the most prominent deformations seen in micrographs. The computational cost of these calculations is fairly low and simplifies the management of boundaries. This approach requires the use of a 4-dimensional transformation operator, i.e., a 2 × 2 matrix. These variables involved can be expressed as the rotation of the structure prior to deformation (ϕ_*R*_), the primary radial deformation (r¯
 MathType@MTEF@5@5@+=feaafiart1ev1aaatCvAUfKttLearuWrP9MDH5MBPbIqV92AaeXatLxBI9gBaebbnrfifHhDYfgasaacH8akY=wiFfYdH8Gipec8Eeeu0xXdbba9frFj0=OqFfea0dXdd9vqai=hGuQ8kuc9pgc9s8qqaq=dirpe0xb9q8qiLsFr0=vr0=vr0dc8meaabaqaciaacaGaaeqabaqabeGadaaakeaacuWGYbGCgaqeaaaa@2E31@), the rate of the deformation giving rise to the elliptical structure (*d*) and the rotation following the deformation (ϕ_*D*_). Together these form the transformation shown below:

T=RDDRR=(cos⁡ϕD−sin⁡ϕDsin⁡ϕDcos⁡ϕD)(r¯d00r¯/d)(cos⁡ϕR−sin⁡ϕRsin⁡ϕRcos⁡ϕR)     (eq. 1)
 MathType@MTEF@5@5@+=feaafiart1ev1aaatCvAUfKttLearuWrP9MDH5MBPbIqV92AaeXatLxBI9gBaebbnrfifHhDYfgasaacH8akY=wiFfYdH8Gipec8Eeeu0xXdbba9frFj0=OqFfea0dXdd9vqai=hGuQ8kuc9pgc9s8qqaq=dirpe0xb9q8qiLsFr0=vr0=vr0dc8meaabaqaciaacaGaaeqabaqabeGadaaakeaacqWGubavcqGH9aqpcqWGsbGudaWgaaWcbaGaemiraqeabeaakiabdseaejabdkfasnaaBaaaleaacqWGsbGuaeqaaOGaeyypa0ZaaeWaceaafaqabeGacaaabaGagi4yamMaei4Ba8Maei4CamNaeqy1dO2aaSbaaSqaaiabdseaebqabaaakeaacqGHsislcyGGZbWCcqGGPbqAcqGGUbGBcqaHvpGAdaWgaaWcbaGaemiraqeabeaaaOqaaiGbcohaZjabcMgaPjabc6gaUjabew9aQnaaBaaaleaacqWGebaraeqaaaGcbaGagi4yamMaei4Ba8Maei4CamNaeqy1dO2aaSbaaSqaaiabdseaebqabaaaaaGccaGLOaGaayzkaaWaaeWaceaafaqabeGacaaabaGafmOCaiNbaebacqWGKbazaeaacqaIWaamaeaacqaIWaamaeaacuWGYbGCgaqeaiabc+caViabdsgaKbaaaiaawIcacaGLPaaadaqadiqaauaabeqaciaaaeaacyGGJbWycqGGVbWBcqGGZbWCcqaHvpGAdaWgaaWcbaGaemOuaifabeaaaOqaaiabgkHiTiGbcohaZjabcMgaPjabc6gaUjabew9aQnaaBaaaleaacqWGsbGuaeqaaaGcbaGagi4CamNaeiyAaKMaeiOBa4Maeqy1dO2aaSbaaSqaaiabdkfasbqabaaakeaacyGGJbWycqGGVbWBcqGGZbWCcqaHvpGAdaWgaaWcbaGaemOuaifabeaaaaaakiaawIcacaGLPaaacaWLjaGaaCzcamaabmGabaGaeeyzauMaeeyCaeNaeeOla4IaeeiiaaIaeeymaedacaGLOaGaayzkaaaaaa@8783@

**Figure 7:**
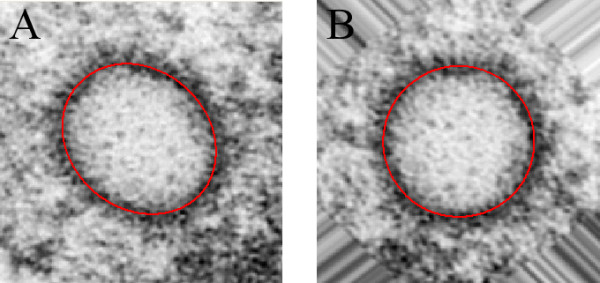
Use of an ellipse to detect linear deformations of virus particles in electron micrographs. Image A has an elliptical shape, whereas image B has been deformed as described to make it circular.

In order to identify the variables of the transformation for an individual virus particle, an ellipse set manually was used to estimate the position, size and deformation of each capsid wall (Figure 7). Thus providing three (ϕ_*D*_, r¯
 MathType@MTEF@5@5@+=feaafiart1ev1aaatCvAUfKttLearuWrP9MDH5MBPbIqV92AaeXatLxBI9gBaebbnrfifHhDYfgasaacH8akY=wiFfYdH8Gipec8Eeeu0xXdbba9frFj0=OqFfea0dXdd9vqai=hGuQ8kuc9pgc9s8qqaq=dirpe0xb9q8qiLsFr0=vr0=vr0dc8meaabaqaciaacaGaaeqabaqabeGadaaakeaacuWGYbGCgaqeaaaa@2E31@ and *d*) of the four variables. The sample was then partially transformed to obtain the primary radius measured without deformation (*d *= 1).

**Figure 8:**
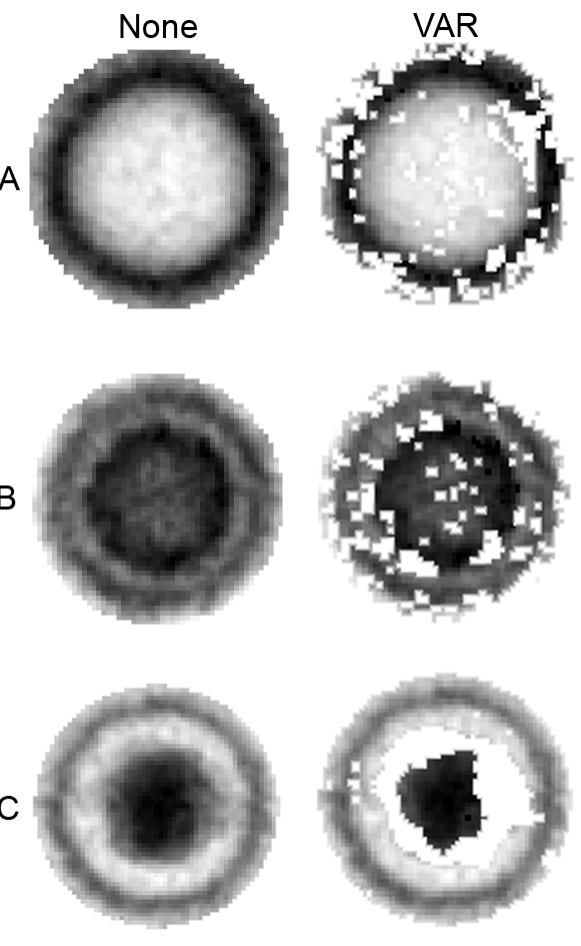
Test functions for viral capsid structures (A, B and C) in electron micrographs employing no coefficient reduction (None) or 80% of the coefficients exhibiting least variation (VAR).

Features that are dependent of rotation such as the polygonal architecture of the capsid wall and position of the DNA core are determined by the ϕ_*R *_value for each sample. In order to find this value, each partially transformed sample was normalized around its mean in the interior of a circle covering the visually significant area of the image (see the images in the left column of Figure 8). Then, the sum of the squares of the distances in the *L*^2^-sence[9] for each sample was minimized with respect to the angles. Since this minimization involves N-1 variables (with N being the number of reference samples considering one sample to be fixed), this procedure was simplified by minimizing the distances to the samples already processed one-by-one. All transformations of the images were implemented in a bi-linear fashion, thereby approximating the value of function *f *at point *(x, y) *as

*f*(*x*, *y*) = *f*(*x*, *y*)(1 - *x*_*m*_)(1 - *y*_*m*_) + *f*(x¯
 MathType@MTEF@5@5@+=feaafiart1ev1aaatCvAUfKttLearuWrP9MDH5MBPbIqV92AaeXatLxBI9gBaebbnrfifHhDYfgasaacH8akY=wiFfYdH8Gipec8Eeeu0xXdbba9frFj0=OqFfea0dXdd9vqai=hGuQ8kuc9pgc9s8qqaq=dirpe0xb9q8qiLsFr0=vr0=vr0dc8meaabaqaciaacaGaaeqabaqabeGadaaakeaacuWG4baEgaqeaaaa@2E3D@, *y*)*x*_*m *_(1 - *y*_*m*_) + *f*(*x*, y¯
 MathType@MTEF@5@5@+=feaafiart1ev1aaatCvAUfKttLearuWrP9MDH5MBPbIqV92AaeXatLxBI9gBaebbnrfifHhDYfgasaacH8akY=wiFfYdH8Gipec8Eeeu0xXdbba9frFj0=OqFfea0dXdd9vqai=hGuQ8kuc9pgc9s8qqaq=dirpe0xb9q8qiLsFr0=vr0=vr0dc8meaabaqaciaacaGaaeqabaqabeGadaaakeaacuWG5bqEgaqeaaaa@2E3F@)(1 - *x*_*m*_)*y*_*m *_+ *f*(x¯
 MathType@MTEF@5@5@+=feaafiart1ev1aaatCvAUfKttLearuWrP9MDH5MBPbIqV92AaeXatLxBI9gBaebbnrfifHhDYfgasaacH8akY=wiFfYdH8Gipec8Eeeu0xXdbba9frFj0=OqFfea0dXdd9vqai=hGuQ8kuc9pgc9s8qqaq=dirpe0xb9q8qiLsFr0=vr0=vr0dc8meaabaqaciaacaGaaeqabaqabeGadaaakeaacuWG4baEgaqeaaaa@2E3D@, y¯
 MathType@MTEF@5@5@+=feaafiart1ev1aaatCvAUfKttLearuWrP9MDH5MBPbIqV92AaeXatLxBI9gBaebbnrfifHhDYfgasaacH8akY=wiFfYdH8Gipec8Eeeu0xXdbba9frFj0=OqFfea0dXdd9vqai=hGuQ8kuc9pgc9s8qqaq=dirpe0xb9q8qiLsFr0=vr0=vr0dc8meaabaqaciaacaGaaeqabaqabeGadaaakeaacuWG5bqEgaqeaaaa@2E3F@)*x*_*m*_*y*_*m*_

where *x*is the nearest smaller integer value of *x*, x¯
 MathType@MTEF@5@5@+=feaafiart1ev1aaatCvAUfKttLearuWrP9MDH5MBPbIqV92AaeXatLxBI9gBaebbnrfifHhDYfgasaacH8akY=wiFfYdH8Gipec8Eeeu0xXdbba9frFj0=OqFfea0dXdd9vqai=hGuQ8kuc9pgc9s8qqaq=dirpe0xb9q8qiLsFr0=vr0=vr0dc8meaabaqaciaacaGaaeqabaqabeGadaaakeaacuWG4baEgaqeaaaa@2E3D@ is the closest higher integer value and *x*_*m *_= *x *- *x*. Integration was performed using the same interpolation. The measurements obtained from this processing step provide indications of the range of the deformation properties, i.e., the main radii (primary radius) and deformation rate, but these parameters should be determined on the basis of additional experience. Since all types of rotation and all directions of deformation of the viral structures are expected to be present in the electron micrographs, these variables were not fixed.

### Identification of points and local function values (parameters) for the virus particle templates

Once the deformed samples are aligned with the partial structure at the same positions, this approach can be used to find the values of the invariant function. In order to describe this procedure more clearly, a deformed sample *f *can be converted into a graph of this function by enumerating (list individually) the pixel positions *x *and their corresponding function values *c *as *f *= {(*x*_*k*_, *c*_*k*_)}_*k*_. The degree of matching between two sequences of function values *y*_*i *_and *y*_*j *_(referred to below as vectors) containing the same sequence of pixel positions was determined using the standard estimated statistical correlation:

M(yi,yj)=〈yi−y¯i,yj−y¯j〉‖yi−y¯i‖‖yj−y¯j‖     (eq. 2a)
 MathType@MTEF@5@5@+=feaafiart1ev1aaatCvAUfKttLearuWrP9MDH5MBPbIqV92AaeXatLxBI9gBaebbnrfifHhDYfgasaacH8akY=wiFfYdH8Gipec8Eeeu0xXdbba9frFj0=OqFfea0dXdd9vqai=hGuQ8kuc9pgc9s8qqaq=dirpe0xb9q8qiLsFr0=vr0=vr0dc8meaabaqaciaacaGaaeqabaqabeGadaaakeaacqWGnbqtcqGGOaakcqWG5bqEdaWgaaWcbaGaemyAaKgabeaakiabcYcaSiabdMha5naaBaaaleaacqWGQbGAaeqaaOGaeiykaKIaeyypa0ZaaSaaaeaacqGHPms4cqWG5bqEdaWgaaWcbaGaemyAaKgabeaakiabgkHiTiqbdMha5zaaraWaaSbaaSqaaiabdMgaPbqabaGccqGGSaalcqWG5bqEdaWgaaWcbaGaemOAaOgabeaakiabgkHiTiqbdMha5zaaraWaaSbaaSqaaiabdQgaQbqabaGccqGHQms8aeaadaqbdiqaaiabdMha5naaBaaaleaacqWGPbqAaeqaaOGaeyOeI0IafmyEaKNbaebadaWgaaWcbaGaemyAaKgabeaaaOGaayzcSlaawQa7amaafmGabaGaemyEaK3aaSbaaSqaaiabdQgaQbqabaGccqGHsislcuWG5bqEgaqeamaaBaaaleaacqWGQbGAaeqaaaGccaGLjWUaayPcSdaaaiaaxMaacaWLjaWaaeWaceaacqqGLbqzcqqGXbqCcqqGUaGlcqqGGaaicqqGYaGmcqqGHbqyaiaawIcacaGLPaaaaaa@6829@

Where y¯
 MathType@MTEF@5@5@+=feaafiart1ev1aaatCvAUfKttLearuWrP9MDH5MBPbIqV92AaeXatLxBI9gBaebbnrfifHhDYfgasaacH8akY=wiFfYdH8Gipec8Eeeu0xXdbba9frFj0=OqFfea0dXdd9vqai=hGuQ8kuc9pgc9s8qqaq=dirpe0xb9q8qiLsFr0=vr0=vr0dc8meaabaqaciaacaGaaeqabaqabeGadaaakeaacuWG5bqEgaqeaaaa@2E3F@ is the mean value of the vector and the matching of all coefficients to [-1,1] is mapped. The justification for using this approach is that it indicates the degree of similarity between the two structures. After placing the sample vectors normalized around their mean y^i=yi−y¯i‖yi−y¯i‖
 MathType@MTEF@5@5@+=feaafiart1ev1aaatCvAUfKttLearuWrP9MDH5MBPbIqV92AaeXatLxBI9gBaebbnrfifHhDYfgasaacH8akY=wiFfYdH8Gipec8Eeeu0xXdbba9frFj0=OqFfea0dXdd9vqai=hGuQ8kuc9pgc9s8qqaq=dirpe0xb9q8qiLsFr0=vr0=vr0dc8meaabaqaciaacaGaaeqabaqabeGadaaakeaacuWG5bqEgaqcamaaBaaaleaacqWGPbqAaeqaaOGaeyypa0ZaaSaaaeaacqWG5bqEdaWgaaWcbaGaemyAaKgabeaakiabgkHiTiqbdMha5zaaraWaaSbaaSqaaiabdMgaPbqabaaakeaadaqbdiqaaiabdMha5naaBaaaleaacqWGPbqAaeqaaOGaeyOeI0IafmyEaKNbaebadaWgaaWcbaGaemyAaKgabeaaaOGaayzcSlaawQa7aaaaaaa@4241@ into columns in a matrix *A*, the test function sequence *f*_*C *_(||*f*_*C*_|| = 1) that makes ||*A*^*T *^*f*_*C*_|| as large as possible is determined, thus providing the best match to the samples used for training. Singular value decomposition (SVD) [10] ||*A*^*T *^*f*_*C*_|| = ||*V*Σ*U*^*T *^*f*_*C*_|| = (V is square and orthonormal) = ||Σ*U*^*T *^*f*_*C*_|| = ||Σ*w*|| is applied to A where ||*w*|| = 1 if *f*_*C *_∈ *span*(*U*) which would be expected. This last expression is maximal when *w *is the eigenvector corresponding to the largest eigenvalue of Σ (which is the largest singular value) and *f*_*C *_should thus be the corresponding column of *U*. Since this function is a linear combination of the columns in *A*, the matching (eq. 2a) reduces to

M(fC,y)=〈fC,y〉‖y−y¯‖     (eq. 2b)
 MathType@MTEF@5@5@+=feaafiart1ev1aaatCvAUfKttLearuWrP9MDH5MBPbIqV92AaeXatLxBI9gBaebbnrfifHhDYfgasaacH8akY=wiFfYdH8Gipec8Eeeu0xXdbba9frFj0=OqFfea0dXdd9vqai=hGuQ8kuc9pgc9s8qqaq=dirpe0xb9q8qiLsFr0=vr0=vr0dc8meaabaqaciaacaGaaeqabaqabeGadaaakeaacqWGnbqtcqGGOaakcqWGMbGzdaWgaaWcbaGaem4qameabeaakiabcYcaSiabdMha5jabcMcaPiabg2da9maalaaabaGaeyykJeUaemOzay2aaSbaaSqaaiabdoeadbqabaGccqGGSaalcqWG5bqEcqGHQms8aeaadaqbdiqaaiabdMha5jabgkHiTiqbdMha5zaaraaacaGLjWUaayPcSdaaaiaaxMaacaWLjaWaaeWaceaacqqGLbqzcqqGXbqCcqqGUaGlcqqGGaaicqqGYaGmcqqGIbGyaiaawIcacaGLPaaaaaa@4E91@

The test function in this initial SVD utilizes the coefficients of all points associated with the first support assumed. Some of these points are located somewhat outside of the viral structures in the images, and in addition, there are points in the structures whose coefficients can vary considerably. Thus, in order to rank the significance of each coefficient and thereby eliminate the worst of the variance, the value of

VARj=∑n=1..N([y^n−〈fC,y^n〉fC]j)2
 MathType@MTEF@5@5@+=feaafiart1ev1aaatCvAUfKttLearuWrP9MDH5MBPbIqV92AaeXatLxBI9gBaebbnrfifHhDYfgasaacH8akY=wiFfYdH8Gipec8Eeeu0xXdbba9frFj0=OqFfea0dXdd9vqai=hGuQ8kuc9pgc9s8qqaq=dirpe0xb9q8qiLsFr0=vr0=vr0dc8meaabaqaciaacaGaaeqabaqabeGadaaakeaacqWGwbGvcqWGbbqqcqWGsbGudaWgaaWcbaGaemOAaOgabeaakiabg2da9maaqafabaWaaeWaceaadaWadiqaaiqbdMha5zaajaWaaSbaaSqaaiabd6gaUbqabaGccqGHsislcqGHPms4cqWGMbGzdaWgaaWcbaGaem4qameabeaakiabcYcaSiqbdMha5zaajaWaaSbaaSqaaiabd6gaUbqabaGccqGHQms8cqWGMbGzdaWgaaWcbaGaem4qameabeaaaOGaay5waiaaw2faamaaBaaaleaacqWGQbGAaeqaaaGccaGLOaGaayzkaaWaaWbaaSqabeaacqaIYaGmaaaabaGaemOBa4Maeyypa0JaeGymaeJaeiOla4IaeiOla4IaemOta4eabeqdcqGHris5aaaa@5213@

was calculated for each coefficient. A certain percentage of the points could then be retained in the test function. Since these operations change on the basis of the test function, a new SVD was subsequently calculated. Figure 8 illustrates the test functions obtained using all coefficients or only those 80% of the varying coefficients identified exhibiting least variance according to the variance ranking. Clearly the size of the DNA core varies in the test function for the C capsid and hence the most uncertain points have been eliminated in the right hand image. Accordingly the test functions obtained by reducing the number of coefficients in this manner were employed routinely.

### Synthesis of the deformations

Since the structures analyzed were assumed to be both oriented in any direction and linearly deformed in any direction, these features must be automatically applied to the test function when analyzing an image. The information provided by the behavior of the matching function when deforming the test function is also of interest for and has been exploited in a similar situation described by Berger et al[11]. While maintaining image *B *and the test function *f*_*C *_fixed and varying the deformation *T*, analysis of the matching function *g*(*T*) = *M *(*f*_*C*_, {*B *(*Tx*_*k*_)}_*k*_) (where the sequence {*x*_*k*_}_*k *_is obtained from the production of the test functions performed. In order to describe *T *in terms of the parameters (φ_*R*_, r¯
 MathType@MTEF@5@5@+=feaafiart1ev1aaatCvAUfKttLearuWrP9MDH5MBPbIqV92AaeXatLxBI9gBaebbnrfifHhDYfgasaacH8akY=wiFfYdH8Gipec8Eeeu0xXdbba9frFj0=OqFfea0dXdd9vqai=hGuQ8kuc9pgc9s8qqaq=dirpe0xb9q8qiLsFr0=vr0=vr0dc8meaabaqaciaacaGaaeqabaqabeGadaaakeaacuWGYbGCgaqeaaaa@2E31@, *d*, φ_*D*_) ∈ ([0,2π], [r¯
 MathType@MTEF@5@5@+=feaafiart1ev1aaatCvAUfKttLearuWrP9MDH5MBPbIqV92AaeXatLxBI9gBaebbnrfifHhDYfgasaacH8akY=wiFfYdH8Gipec8Eeeu0xXdbba9frFj0=OqFfea0dXdd9vqai=hGuQ8kuc9pgc9s8qqaq=dirpe0xb9q8qiLsFr0=vr0=vr0dc8meaabaqaciaacaGaaeqabaqabeGadaaakeaacuWGYbGCgaqeaaaa@2E31@_0_, r¯
 MathType@MTEF@5@5@+=feaafiart1ev1aaatCvAUfKttLearuWrP9MDH5MBPbIqV92AaeXatLxBI9gBaebbnrfifHhDYfgasaacH8akY=wiFfYdH8Gipec8Eeeu0xXdbba9frFj0=OqFfea0dXdd9vqai=hGuQ8kuc9pgc9s8qqaq=dirpe0xb9q8qiLsFr0=vr0=vr0dc8meaabaqaciaacaGaaeqabaqabeGadaaakeaacuWGYbGCgaqeaaaa@2E31@_1_], [*d*_0_, *d*_1_], [0,2π]) = *T*_*bound*_, the following assumptions are made:

*(i) *For certain *T *∈ *T*_*bound*_, the deformed test function represents the structure most similar to the object in the image. It is assumed that this *T *is the one that maximizes *g*.

*(ii) *The *T *associated with the maximal deformation should be localized within the interior of the deformation set, and not on the boundary. Under these conditions, even if *g *is maximized outside the set (i.e. the structure is too large, too small or too badly deformed), matching with the nearest boundary points could still be high.

To be considered identified, a structure should match these criteria. Maximization of the matching function was performed with a reversed steepest descent scheme[12], using the non-deformed test function as a starting point and approximating the derivative as an eight-point, centered difference scheme (i.e. two points for each variable in the deformation).

**Figure 9:**
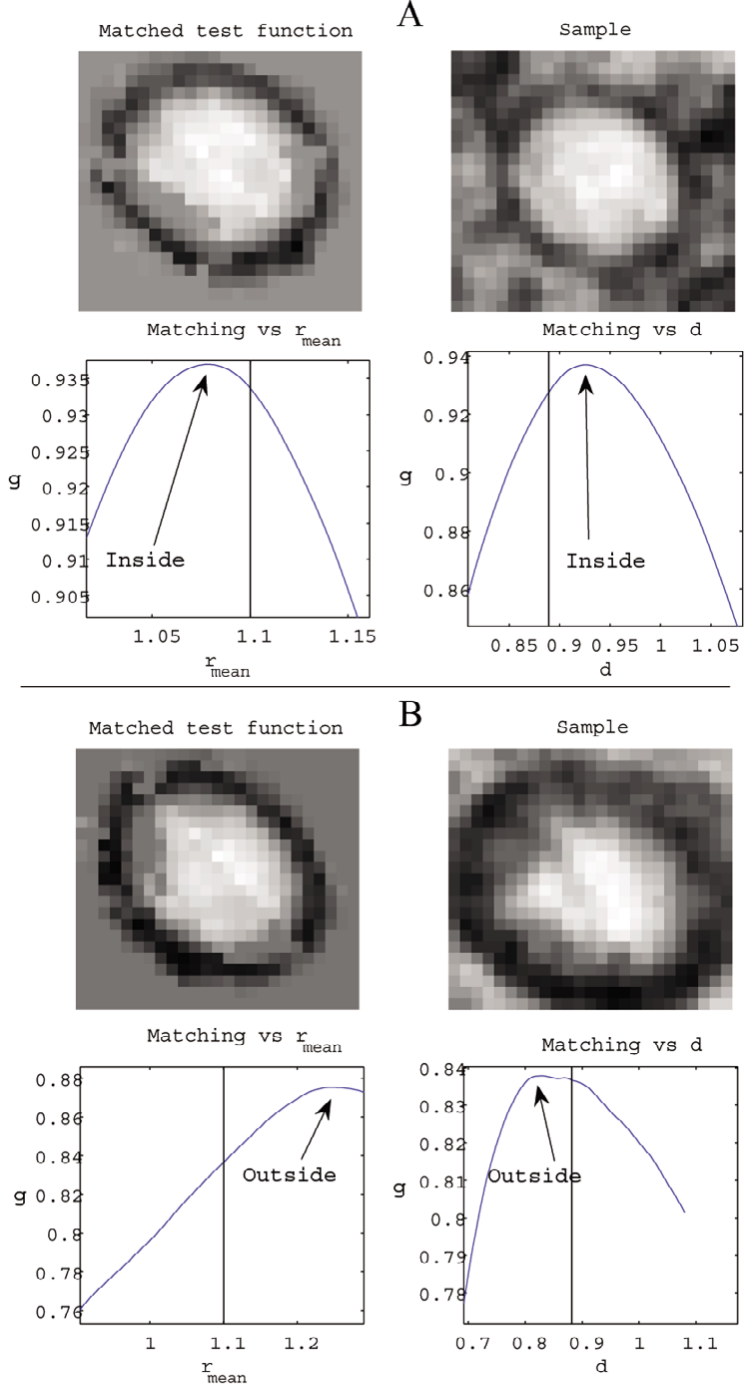
Matching of the test function A to an authentic capsid structure, as well as to a similar but false structure. (A) An authentic capsid image. When the test function is deformed, the graphs illustrates how the matching function *g *varies with radial size (r¯
 MathType@MTEF@5@5@+=feaafiart1ev1aaatCvAUfKttLearuWrP9MDH5MBPbIqV92AaeXatLxBI9gBaebbnrfifHhDYfgasaacH8akY=wiFfYdH8Gipec8Eeeu0xXdbba9frFj0=OqFfea0dXdd9vqai=hGuQ8kuc9pgc9s8qqaq=dirpe0xb9q8qiLsFr0=vr0=vr0dc8meaabaqaciaacaGaaeqabaqabeGadaaakeaacuWGYbGCgaqeaaaa@2E31@) and degree of deformation (*d*}) from the point in the set of admissible deformations that maximizes *g*. The deformed test function has an appearance similar to that of the sample, and the deformation is inside the boundaries. The classification should thus be positive. (B) Unlike (A), the point in the deformation set that maximizes g is situated on the boundary and the graphs show a higher matching value outside of this set. Thus, this classification should be negative.

**Figure 10:**
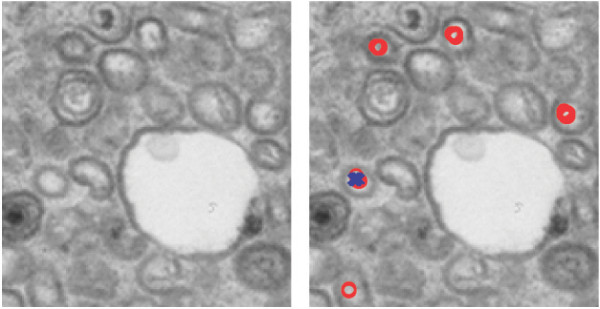
Matching with the test function A inside of a vesicle. The structure marked with a blue cross fulfills matching criteria *(i) *and *(ii) *whereas those marked with a red circle only fulfill criterion *(i)*.

Application of the matching criteria employed is depicted in Figures 9 and 10. Figure 9 illustrates how these criteria work when applied to an authentic A capsid, as well as to a similar but false structure. In this case the deformation boundaries were set to (φ_*R*_, r¯
 MathType@MTEF@5@5@+=feaafiart1ev1aaatCvAUfKttLearuWrP9MDH5MBPbIqV92AaeXatLxBI9gBaebbnrfifHhDYfgasaacH8akY=wiFfYdH8Gipec8Eeeu0xXdbba9frFj0=OqFfea0dXdd9vqai=hGuQ8kuc9pgc9s8qqaq=dirpe0xb9q8qiLsFr0=vr0=vr0dc8meaabaqaciaacaGaaeqabaqabeGadaaakeaacuWGYbGCgaqeaaaa@2E31@, *d*, φ_*D*_) ∈ ([0,2π], [0.89,1.1], [0.89,1.13], [0,2π]) for illustrative purposes.

Viral capsids exit the nucleus by budding through the membrane of this organelle. In connection with this process it is difficult to discriminate between viral and other structures, as shown in Figure 10. In this figure a blue cross indicates a point in the image where the match between the test function and the capsid structure match is better than 0.8 and the degree of deformation is acceptable. A red circle indicates a point at which this match is better than 0.8, but where the degree of deformation is not admissible. The structure marked as a match has a matching of 0.94, which is very high.

### Identification of virus particle structures in an electron microscopic image

In order to search for structures in an image *B *similar to the test function *f*_*C*_, eq 2b is expanded to convolutions. The matching of the test function at a point *m *can thus be expressed as

MB,fC(m)=sup⁡T∈TboundM(fC,{B(m+Txk)}k)
 MathType@MTEF@5@5@+=feaafiart1ev1aaatCvAUfKttLearuWrP9MDH5MBPbIqV92AaeXatLxBI9gBaebbnrfifHhDYfgasaacH8akY=wiFfYdH8Gipec8Eeeu0xXdbba9frFj0=OqFfea0dXdd9vqai=hGuQ8kuc9pgc9s8qqaq=dirpe0xb9q8qiLsFr0=vr0=vr0dc8meaabaqaciaacaGaaeqabaqabeGadaaakeaacqWGnbqtdaWgaaWcbaGaemOqaiKaeiilaWIaemOzay2aaSbaaWqaaiabdoeadbqabaaaleqaaOGaeiikaGIaemyBa0MaeiykaKIaeyypa0ZaaCbeaeaacyGGZbWCcqGG1bqDcqGGWbaCaSqaaiabdsfaujabgIGiolabdsfaunaaBaaameaacqWGIbGycqWGVbWBcqWG1bqDcqWGUbGBcqWGKbazaeqaaaWcbeaakiabd2eanjabcIcaOiabdAgaMnaaBaaaleaacqWGdbWqaeqaaOGaeiilaWIaei4EaSNaemOqaiKaeiikaGIaemyBa0Maey4kaSIaemivaqLaemiEaG3aaSbaaSqaaiabdUgaRbqabaGccqGGPaqkcqGG9bqFdaWgaaWcbaGaem4AaSgabeaakiabcMcaPaaa@5A58@

However, this procedure is highly time-consuming. It can be accelerated by making a few observations and assumptions:

*(i) *The deformed variants of the test functions are not orthogonal to one another, and because these structures are essentially independent of rotation, the match of the non-deformed test function is better than that of a certain value to any admissible deformed structure of the same kind.

*(ii) *Since translation deforms a structure further, matching to the non-deformed test function is assumed to be higher at the actual position of a virus particle than at locations at least one diameter of the test function distant from this position.

Implementing these criteria, one can identify a subset of potentially interesting points within the larger image. Thereafter further analysis of this set employing the optimization described in the preceding section can be performed. This approach provides a final set of points in the image that are associated with matching values of *P *= {*M*_*j*_}_*j*_. In order to ensure inclusion of all interesting positions in an image the threshold value connected with assumption *(i) *above was set to 0.5.

### Post-processing of the final set: counting virus particles

There is no threshold value *t *that can distinguish between authentic and false structures in all images, i.e., the assignment of structures employing this procedure does not agree completely with that done by an experienced virologist. Setting a threshold level is therefore not an option. Instead, a positive probability function *PPF *: [-1,1] → [0,1] can be used to determine the probability that a given point associated with a certain matching value is actually associated with the virus particle. This extension of the positive predictive value (PPV) is obtained by calculating the ratio between the number of correctly identified structures and the total number of structures identified with a certain matching value. Thus, for a set *P *of structures identified by this procedure containing the subset *P*_*correct *_of points associated with virus particles of a given kind,

PPF(M)=#{Mk∈Pcorrect;M≤Mk<M+ε}#{Mk∈P;M≤Mk<M+ε}.
 MathType@MTEF@5@5@+=feaafiart1ev1aaatCvAUfKttLearuWrP9MDH5MBPbIqV92AaeXatLxBI9gBaebbnrfifHhDYfgasaacH8akY=wiFfYdH8Gipec8Eeeu0xXdbba9frFj0=OqFfea0dXdd9vqai=hGuQ8kuc9pgc9s8qqaq=dirpe0xb9q8qiLsFr0=vr0=vr0dc8meaabaqaciaacaGaaeqabaqabeGadaaakeaacqWGqbaucqWGqbaucqWGgbGrcqGGOaakcqWGnbqtcqGGPaqkcqGH9aqpdaWcaaqaaiabcocaJiabcUha7jabd2eannaaBaaaleaacqWGRbWAaeqaaOGaeyicI4Saemiuaa1aaSbaaSqaaiabdogaJjabd+gaVjabdkhaYjabdkhaYjabdwgaLjabdogaJjabdsha0bqabaGccqGG7aWocqWGnbqtcqGHKjYOcqWGnbqtdaWgaaWcbaGaem4AaSgabeaakiabgYda8iabd2eanjabgUcaRiabew7aLjabc2ha9bqaaiabcocaJiabcUha7jabd2eannaaBaaaleaacqWGRbWAaeqaaOGaeyicI4SaemiuaaLaei4oaSJaemyta0KaeyizImQaemyta00aaSbaaSqaaiabdUgaRbqabaGccqGH8aapcqWGnbqtcqGHRaWkcqaH1oqzcqGG9bqFaaGaeiOla4caaa@679D@

In order to obtain a smooth and monotonically increasing function 0.05 was chosen as the value for ε. The probability function indicating the expected number *N *of structures in the image is E(N)=∑M∈PPPF(M)
 MathType@MTEF@5@5@+=feaafiart1ev1aaatCvAUfKttLearuWrP9MDH5MBPbIqV92AaeXatLxBI9gBaebbnrfifHhDYfgasaacH8akY=wiFfYdH8Gipec8Eeeu0xXdbba9frFj0=OqFfea0dXdd9vqai=hGuQ8kuc9pgc9s8qqaq=dirpe0xb9q8qiLsFr0=vr0=vr0dc8meaabaqaciaacaGaaeqabaqabeGadaaakeaacqWGfbqrcqGGOaakcqWGobGtcqGGPaqkcqGH9aqpdaaeqbqaaiabdcfaqjabdcfaqjabdAeagjabcIcaOiabd2eanjabcMcaPaWcbaGaemyta0KaeyicI4SaemiuaafabeqdcqGHris5aaaa@3DCB@.

### Accuracy of the method

In order to organize viral particles seen in electron micrographs according to their stage of maturation, a model such as that described here, is required to portray each particular stage. Furthermore, for this model to be useful for the detection and quantization of virus particles in such images it must also be able to reject spurious structures. Thus, an ideal model should detect all possible images of virus particles of different kinds, but nothing else located in the same space, i.e., in the background. In order to characterize our model in this respect the commonly false negative (FNR) and false positive (FPR) ratios were utilized. The FNR is defined as the ratio between the number of authentic virus particles rejected incorrectly by the method and the actual number of authentic particles, while The FPR is the ratio between the number of spurious structures identified as being authentic and the total number of structures considered to be authentic by this approach. Thus, both of these ratios lie between 0 and 1, with 0 being ideal.

In order to determine the number of virus particles on the basis of the information provided by the set of matching values acquired by searching through an image, the positive probability function *PPF *described under the Materials and Methods section was utilized. The expected number of particles identified was compared with the true number of particles present in the image to obtain a mean and standard deviation of the counting error. In addition, to evaluate whether there was a systematic mean difference, i.e., whether the procedure identifies on the average too many or too few particle, the H_0 _hypothesis that: "The mean difference = 0" was tested.

## Abbreviations

FNR – false negative ratio

FPR – false positive ratio

HCMV – Human Cytomegalovirus

PPF – positive probability function

PPV – positive predictive value

SVD – Singular value decomposition

## Competing interests

MR and MHL hold, and are currently applying for, patents relating to the content of this manuscript. The authors have received reimbursement from the centre for molecular medicine, department of medicine at the Karolinska Institutet to patent the methodology. MHL is owner of the company intelligent virus imaging AB  that is in the process of commercialising this methodology.

## Authors' contributions

This paper is the result of a thesis work by MR supervised by MHL with the intellectual aid ang guidance of JOS and CSN. This method is a follow up of the work initiated by MHL and Dr. Ida-Maria Sintorn. MHL acquired electron micrographs for analysis and designed the study. Analysis, interpretation of data and method development was done by MR and MHL. MR drafted the layout for this manuscript. MHL revised, wrote and completed it critically for important intellectual content. JOS intellectual contributions was in the process of method development and CSN intellectual contributions was in the analysis and interpretation of data and its implications for use in virus assembly studies.
